# Preoperative Vascular and Cranial Nerve Imaging in Skull Base Tumors

**DOI:** 10.3390/cancers17010062

**Published:** 2024-12-28

**Authors:** Akinari Yamano, Masahide Matsuda, Eiichi Ishikawa

**Affiliations:** Department of Neurosurgery, Institute of Medicine, University of Tsukuba, Tsukuba 305-8575, Japan

**Keywords:** skull base tumor, schwannoma, meningioma, vascular, cranial nerve, preoperative imaging

## Abstract

Skull base tumors such as meningiomas and schwannomas are often pathologically benign. However, surgery for these tumors poses significant challenges because of their proximity to critical structures such as the brainstem, cerebral arteries, veins, and cranial nerves. These structures are compressed or encased by the tumor as they grow, increasing the risk of unintended injury to these structures, which can potentially lead to severe neurological deficits. Preoperative imaging is crucial for assessing the tumor size, location, and its relationship with adjacent vital structures. This study reviews advanced imaging techniques that allow detailed visualization of vascular structures and cranial nerves. Contrast-enhanced computed tomography and digital subtraction angiography are optimal for evaluating vascular structures, whereas magnetic resonance imaging (MRI) with high-resolution T2-weighted images and diffusion tensor imaging are optimal for evaluating cranial nerves. These methods help surgeons plan tumor resection strategies, including surgical approaches, more precisely. An accurate preoperative assessment can contribute to safe tumor resection and preserve neurological function. Additionally, we report the MRI contrast defect sign in skull base meningiomas, which suggests cranial nerve penetration through the tumor. This is an essential finding for inferring the course of cranial nerves completely encased within the tumor. These preoperative imaging techniques have the potential to improve the outcomes of patients with skull base tumors. Furthermore, this study highlights the importance of multimodal imaging approaches and discusses future directions for imaging technology that could further develop preoperative surgical simulations and improve the quality of complex skull base tumor surgeries.

## Simple Summary

Surgery of skull base tumors presents significant challenges for neurosurgeons owing to their proximity to critical structures, such as the brainstem, arteries, veins, and cranial nerves. These tumors are located in deep and narrow intracranial spaces, and the anatomical variations in the surrounding structures differ among patients. Consequently, complete tumor resection carries the risk of damaging these vital structures, potentially leading to severe neurological deficits. Preoperative imaging plays a crucial role in assessing tumors and their relationships with adjacent structures. This study reviewed advanced imaging techniques that allow detailed visualization of important structures using different modalities such as computed tomography, magnetic resonance imaging (MRI), and digital subtraction angiography. Moreover, we report the MRI contrast defect sign, which suggests that the cranial nerve penetrates the skull base meningiomas. These methods improve the accuracy of preoperative assessment, guide surgical planning, reduce complications, and preserve neurological functions.

## 1. Introduction

Among skull base tumors treated by neurosurgeons, the majority are histologically benign, such as meningiomas and schwannomas. It is established that the extent of tumor resection significantly influences long-term postoperative tumor control [1,2,3,4,5]. However, these tumors are located in deep and narrow intracranial spaces, frequently adjacent to critical structures, such as the brainstem, arteries, veins, and cranial nerves. As the tumor grows, these structures are compressed or encased. Pathological anatomical variations in these structures differ significantly among patients. Therefore, radical resection carries the risk of deteriorating the patient’s function and quality of life [6,7,8,9,10]. For surgical support, neuromonitoring and neuronavigation are useful to prevent injury to surrounding structures [11,12,13,14]. Moreover, preoperative imaging assessment is crucial for ensuring the safety of surgery, as well as for efficient tumor resection. It is necessary to assess the size and location of the tumor, as well as its anatomical relationship with adjacent structures, such as the cerebral vessels and cranial nerves. This information is valuable for determining the surgical approach and planning tumor resection strategies.

For preoperative tumor evaluation, contrast-enhanced computed tomography (CT), magnetic resonance imaging (MRI), and digital subtraction angiography (DSA) are often used. Recent advances in these modalities have made it possible to obtain more detailed preoperative tumor information using less invasive methods. Here, we review methods for preoperatively identifying the surrounding structures in skull base tumors. We summarize the previous literature on the evaluation of vascular structures and cranial nerves related to skull base tumors and introduce methods for cranial nerve visualization based on our experience.

## 2. Visualization of Vascular Structures with Skull Base Tumors

Preoperative vascular assessment during skull base tumor surgery is necessary to avoid complications associated with tumor resection and surgical approaches. Vascular injury associated with tumor resection is one of the most critical complications to avoid, and the normal arteries running near or through the tumor should be evaluated preoperatively [15,16,17,18]. Evaluation of tumor-feeding arteries is useful for assessing tumor vascularity and determining the indications for preoperative embolization [19,20]. Additionally, preserving important veins during tumor resection is essential because it influences decisions regarding the extent of resection and surgical approaches [21,22].

**Figure 1:**
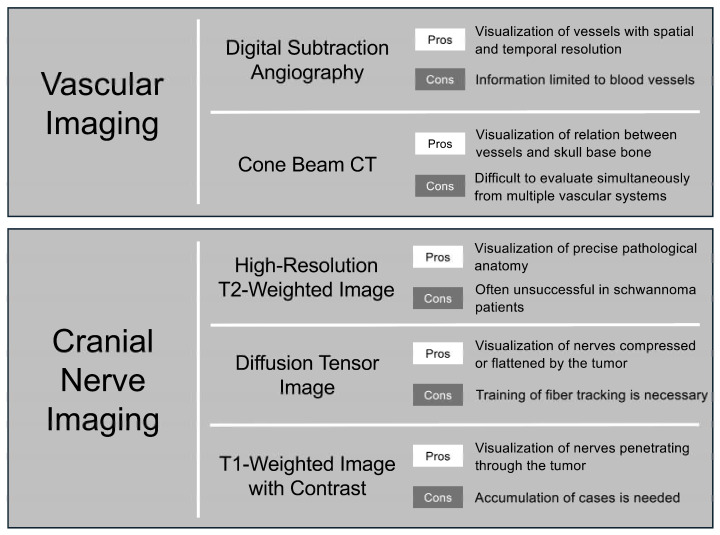
Modality selection and its advantages and disadvantages in preoperative vascular and cranial nerve imaging.

Because contrast agents flow through vessels, vascular structures can be visualized in great detail. By utilizing this information, the exact location of the tumor and its relationship with surrounding vessels can be determined precisely [23,24,25]. Although DSA is more invasive than other modalities, it offers many advantages, making it the gold standard for preoperative vascular evaluation. DSA has high spatial and temporal resolutions, allowing for the visualization of small feeders or surrounding vessels [26]. High-resolution cone beam CT images reconstructed from three-dimensional rotational angiography enable detailed anatomical assessment. With cone beam CT, multiplanar reconstruction of the axial, coronal, and sagittal planes allows for more accurate and comprehensive information acquisition. The relationship between the skull base bone and tumor-feeding arteries or small perforators running near the tumor can be visualized using cone beam CT [20,27]. In addition, DSA can visualize vessels from each arterial system separately, and high-risk pial feeders from the internal carotid artery or vertebral artery systems can be highlighted compared with other modalities (Figure 1) [20]. In addition, DSA enables the assessment of tumor blood flow, which helps evaluate surgical risks and determine the indications for preoperative tumor embolization [19]. There has been controversy regarding the benefits of preoperative feeding artery embolization for skull base meningiomas [28]. However, in selected cases, feeder embolization before tumor resection can be a highly useful adjuvant therapy [20,29].

Skull base tumors sometimes compress or involve venous structures, and injury or sacrifice of venous structures can result in severe complications [30,31,32,33]. Moreover, normal venous drainage may need to be sacrificed when using a specific skull base approach. Because sacrificing the main venous drainage can potentially lead to significant complications, surgical approaches for skull base tumors require preoperative assessment of normal venous drainage courses [21,22,25]. For the evaluation of venous structures, CT or MRI with contrast has been mainly used in previous studies. In addition to the convenience of CT and MRI, evaluating venous structures does not require a detailed assessment of arteries. DSA, with its temporal resolution, may be the best modality for some tumors for which a venous drainage assessment is necessary.

Identifying the location of venous sinuses is important when determining the keyhole position for craniotomy. Surface landmarks on the bone have long been widely used to determine this position, with reports supporting its effectiveness [34,35]. However, variations exist in the relationship between these landmarks, and the actual position of the venous sinuses varies. Therefore, minimizing discrepancies using preoperative imaging is an effective strategy to ensure an accurate and safe craniotomy. The development of neuronavigation systems has made it possible to verify preoperative images in the surgical field during surgery [36], but it is sometimes less reliable in the posterior fossa. It is also possible to reconstruct preoperative information into three-dimensional (3D) simulation data, including the bone, venous sinuses, and other structures [15,37,38]. To ensure safe and effective surgery, it is essential to utilize preoperative imaging information effectively using different methods.

## 3. Visualization of Normal Anatomy of Cranial Nerves

Unlike vascular structures, normal cranial nerves are not well enhanced by contrast agents. Visualization of fine intracranial structures, including cranial nerves, initially began with cisternography using high-resolution CT with contrast agents or gases [39,40]. Subsequently, advancements in MRI have made it possible to visualize these structures noninvasively. Currently, MRI-based visualization of intracranial microanatomy is the gold standard, and several different sequences are available for this purpose. Initially, high-resolution T1-weighted images were used to visualize cranial nerves that were not surrounded by cerebrospinal fluid (CSF). Using the contrast-enhanced technique, T1-weighted images show favorable results in the visualization of nerves surrounded by soft tissue [41]. However, T2-weighted images have a higher contrast between the cranial nerves and CSF than T1-weighted images. Therefore, T2-weighted images have frequently been used to identify the cisternal segments of the cranial nerves surrounded by the CSF. MRI cisternography using high-resolution T2-weighted images has been frequently reported, including driven equilibrium (DRIVE) [42], constructive interference of steady-state sequence (CISS) [43], fast imaging employing steady-state acquisition (FIESTA) [44], fast asymmetric spin echo (FACE), and balanced fast field echo (bFFE) [45]. These methods reduce the CSF-compensated artifacts caused by motion and susceptibility owing to local magnetic field inhomogeneity.

In contrast, the cranial nerves running through the venous sinuses or plexuses can be identified using contrast-enhanced MRI [46,47]. Because normal cranial nerves are not enhanced by the contrast agent, the contrast-filled venous structures allow non-enhanced structures to be identified as cranial nerves. In addition, high-resolution T2-weighted images with intravenous contrast are used to identify cranial nerves in both venous structures and subarachnoid cisterns [47,48]. This technique is valid for visualizing the lower cranial nerves in and out of the jugular foramen and the abducens nerve in the petroclival segment.

The identification of cranial nerves using diffusion tensor imaging (DTI) was conducted using an approach different from the previously mentioned methods. This method uses the unequal diffusion of water molecules along white fibers to extract their direction and then reconstruct the white fiber tracts [49]. Tracking cranial nerves is limited because of their small size [50], tortuous trajectories in skull base cisterns or dura folds, and splayed fibers within bundles [51]. The tracking parameters should be tailored to the anatomical features of each cranial nerve. In addition, an anatomical reference is needed for accurate fiber tracking owing to the low resolution of diffusion imaging. A successful cranial nerve tractography requires experience in anatomy, radiology, and computer science. Therefore, tractography training is necessary to ensure the reliability and reproducibility of the results [52].

Generally, for cranial nerve visualization using DTI, nerves with a thick diameter, straight course, and minimal artifacts from the surrounding structures are optimal for fiber tracking. The nerves adjacent to the bone or paranasal sinuses are highly affected by artifacts. Therefore, the tracking parameters of DTI should be tailored to each nerve considering the anatomical specificities of each cranial nerve. Therefore, the trigeminal and facial nerves are relatively easy to visualize. However, because the fibers of these nerves are thin and diffuse after exiting the brainstem, visualization of the lower cranial nerves is challenging [51]. A higher-resolution DTI scan with a smaller slice thickness could be one of the solutions to this problem; however, it has not been utilized in actual clinical practice.

## 4. Visualization of Cranial Nerves with Skull Base Tumors

The preoperative visualization of cranial nerves is even more challenging in the presence of tumors. Cranial nerves are often compressed or flattened by tumors and sometimes have preoperatively impaired nerve function. This review focuses on meningiomas and schwannomas, which are commonly encountered benign skull base tumors.

### 4.1. Meningiomas

Meningiomas are the most prevalent primary intracranial tumors, accounting for approximately 37% of all intracranial tumors [53]. Meningiomas originate in the meninges and occur at various locations. Surgical resection is the primary treatment for symptomatic or enlarging meningiomas, and the extent of resection is an important prognostic factor [54]. The surgical strategy varies significantly depending on the tumor location, size, vascularity, and adhesion to the surrounding structures. In some cases, the tumor severely compresses the cranial nerves, or the cranial nerves may penetrate the tumor [6]. These features increase the risk of postoperative cranial nerve dysfunction, and efficient tumor resection requires advanced surgical strategies and techniques. Therefore, preoperative visualization of the cranial nerves surrounding the tumor is believed to contribute to improved surgical outcomes and various studies have been conducted on this subject (Table 1).

For parasellar meningiomas such as anterior clinoidal meningiomas, planum sphenoidale meningiomas, and tuberculum sellae meningiomas, preoperative simulation of the relationship between the tumor and the optic nerve is crucial. A report using conventional spin echo T1-weighted imaging and spoiled gradient recalled acquisition in steady state (SPGR) showed that the detection rates of the optic nerve and chiasma were only 28.6% and 50.0% [55]. These T1-weighted images were used to detect the optic nerves in patients with pituitary neuroendocrine tumors. Because meningiomas usually show iso-intensity on T1-weighted images and often have irregular margins compared to pituitary neuroendocrine tumors, the detection rate in the optic nerve declines. Furthermore, optic nerves near the optic canal tend to be difficult to identify in meningiomas of midline origin. However, the detection rate improved when heavily T2-weighted images were used (85.7%) [56]. On T2-weighted images, changes in the intensity of the optic nerve and chiasma due to tumor compression may affect detection. Although the number of cases was limited, one study successfully visualized the optic nerve using DTI tracking. In three cases, 3D reconstruction images based on DTI tracking matched the intraoperatively confirmed course of the optic nerve (100%) [57,58].

For posterior fossa meningiomas, such as petrous, petroclival, and petrotentorial meningiomas, many critical structures are present within a confined space. The trochlear, trigeminal, abducens, facial, vestibulocochlear, lower cranial, and hypoglossal nerves are associated with tumors. High-resolution T2-weighted images without contrast sometimes have difficulty in visualizing cranial nerves compressed by posterior fossa meningiomas [8,59]. However, with contrast-enhanced imaging, cranial nerve pathways were identified with a high degree of accuracy (87.5%) [60]. Meningiomas often demonstrate homogenous enhancement in contrast-enhanced studies. In the FIESTA sequence with contrast, homogenous enhancement of meningiomas and hyperintensity of the CSF further accentuated the boundary with the cranial nerves, demonstrated as hypointense structures. DTI fiber tracking has also been applied to posterior fossa meningiomas. Despite the tumor compression, cranial nerves were successfully visualized in all 15 cases in the previous reports [52,57,58,61,62,63,64]. These reports focused on the visualization of cranial nerves compressed or flattened by meningiomas, and the consideration of cranial nerves penetrating through the tumor has not yet been reported (Figure 1).

### 4.2. Schwannomas

Intracranial schwannomas are the third most common intracranial non-malignant tumors after meningiomas and pituitary neuroendocrine tumors [53]. Most intracranial schwannomas are vestibular schwannomas (95.5%), and most published studies have focused on vestibular schwannomas. Trigeminal and jugular foramen schwannomas account for approximately 2% of intracranial schwannomas and may sometimes be treated as cerebellopontine angle tumors [65,66]. Treatment options for vestibular schwannomas include surgery and stereotactic radiosurgery. Both methods have shown good outcomes in tumor control; however, surgery is preferred for larger tumors and younger patients [67,68,69]. A combination of surgery and radiosurgery has been selected as a surgical strategy for large vestibular schwannomas [70]. The extent of resection is known to affect postoperative tumor outcomes; however, aggressive resection may increase the risk of facial nerve palsy or hearing loss due to damage to the adjacent facial and cochlear nerves [69]. Resection using neuromonitoring is effective in avoiding irreversible nerve injury; however, preoperative assessment of the facial nerve pathway is crucial for safe and effective surgery [71,72].

Table 2 summarizes previous reports on the preoperative imaging visualization of cranial nerves in patients with intracranial schwannomas. While most of the literature focuses on vestibular schwannomas [57,58,59,60,62,63,64,73,74,75,76,77,78,79,80], a few reports include trigeminal and jugular foramen schwannomas [60,62,64]. High-resolution T2-weighted imaging with or without contrast, which demonstrated accurate cranial nerve visualization in patients with meningioma, had a significantly lower cranial nerve identification rate in patients with schwannoma (20%; 9 of 45 patients). The course of the facial nerves has a variety of anatomical patterns in patients with vestibular schwannoma [10]. As the tumor size increases, the facial and cochlear nerves are often stretched over the tumor and are difficult to identify morphologically using MRI. Instead, many reports have used DTI for cranial nerve visualization, proving its high accuracy.

Owing to the incidence of schwannomas, most previous studies have focused on vestibular schwannomas and the facial nerves. Visualization of the facial nerve using DTI is a highly reliable examination that shows a high rate of concordance with intraoperative findings. Large tumor size, cystic tumors, and similar tumor diffusion signals to cranial nerves have been reported as reasons for the inability to perform facial nerve tracking in vestibular schwannomas [74,81]. Visualization of the trigeminal nerves in trigeminal schwannomas has been reported to have a relatively low accuracy [62]. Only one patient with jugular foramen schwannoma had successful DTI tracking of the lower cranial nerves [60]. Devising the target selection of the region of interest, the combined use of DTI and other high-resolution T2-weighted sequences has been reported to improve the accuracy of predicting the course of the facial and cochlear nerves [52,76]. There has been a report of improved facial nerve visualization through the reconstruction of high-resolution DTI; however, comparisons with actual intraoperative findings have not been made [61]. Methods for tractography reconstruction have also been studied, and better accuracy has been achieved compared to standard single-diffusion tractography [64]. However, single-diffusion tractography is still more commonly used clinically because of its high prevalence (Figure 1).

## 5. Cranial Nerves Penetrating Skull Base Tumors

In skull base meningiomas, tumors sometimes completely encase surrounding structures, such as the cranial nerves penetrating the tumor [6]. Previously described imaging techniques assess the course of the cranial nerves adjacent to the tumors, and few reports have focused on cranial nerves completely encased by the tumors. When a tumor completely encases the cranial nerves, careful manipulation is required during surgery to avoid cranial nerve damage. Therefore, preoperative imaging to assess whether the cranial nerves penetrate the tumor could be useful for improving the safety of skull base meningioma surgery. Compared with tumors or vessels, cranial nerves are less enhanced by contrast. Cranial nerves that penetrate the venous sinus, venous plexuses, or uniformly enhanced tumors may appear as linear contrast defects on contrast-enhanced MRI [46,47,82]. We aimed to clarify the relationship between tumor contrast defects observed on preoperative MRI and the actual course of the cranial nerves.

We investigated 68 patients who underwent surgery for posterior fossa meningioma at our institute between January 2017 and December 2023. Preoperative contrast-enhanced MRI was performed in all the cases. To assess the presence of tumor contrast defects of the cranial nerves, a T1-weighted fast-field echo with water-selective excitation (T1 FFE WATS) was performed with a slice thickness of 1.0 mm. The images were scanned in an axial section. We focused on the trigeminal, abducens, lower cranial, and hypoglossal nerves and the facial and vestibulocochlear nerve complexes. The cochlear nerve was excluded owing to its small diameter. The medical records and surgical videos of patients with cranial nerve contrast defects were reviewed retrospectively. Two board-certified neurosurgeons determined the presence of contrast defects and intraoperative findings of the cranial nerves. Disagreements were resolved by consensus.

Contrast defects were identified in 14 cranial nerves in 10 patients (Table 3). Among them, 11 cranial nerves were verified during surgery, all of which penetrated the tumor. We could not confirm the course of the three cranial nerves (two abducens nerves and one facial and vestibulocochlear nerve complex) because of the difficulty in tumor resection. The trigeminal, abducens, and lower cranial nerves were penetrated in five, two, and four patients, respectively. Most patients had benign pathological findings, except for one patient with a clear cell meningioma.

**Figure 2:**
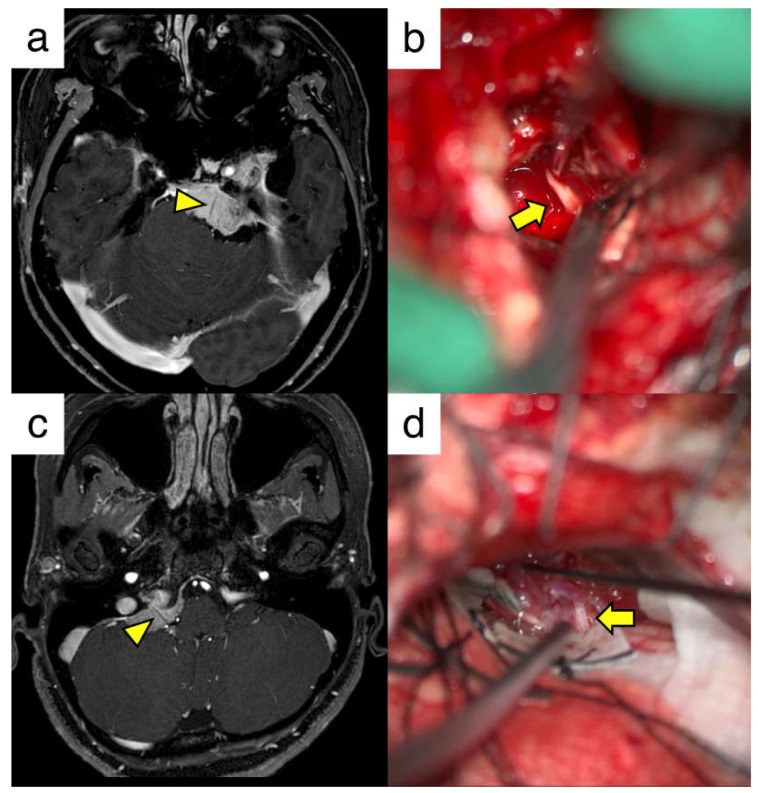
Illustrative cases of skull base meningiomas with cranial nerve penetration. (**a**) T1-weighted image with contrast of a patient with a left petroclival meningioma. The yellow arrowhead shows the contrast defect in the abducens nerve. (**b**) Intraoperative view of the abducens nerve penetrating the tumor (yellow arrow). (**c**) T1-weighted image showing the contrast of the patient with a right petrous meningioma. The yellow arrowhead indicates a contrast defect in the lower cranial nerve. (**d**) Intraoperative view of the glossopharyngeal nerve penetrating through the tumor (yellow arrow).

Here, two illustrative cases are presented. The first case was case number nine with a left petroclival meningioma. A linear contrast defect of the left abducens nerve was observed on the preoperative MRI (Figure 2a). Tumor resection was performed using the anterior transpetrosal approach, and the abducens nerve penetrating the tumor was confirmed during surgery (Figure 2b). Most of the tumor, except for that in the cavernous sinus, was removed, and the function of the abducens nerve was preserved. The second case was case number ten with a right petrous meningioma. Contrast defects were observed in the right lower cranial nerves (Figure 2c). Tumor resection was performed using the lateral suboccipital approach. Tumor penetration of the lower cranial nerve (glossopharyngeal nerve) was verified intraoperatively, and near-total resection of the tumor was achieved (Figure 2d). No postoperative deficits were observed in the penetrating cranial nerves. We believe that preoperative anticipation of cranial nerve tumor penetration contributed to the avoidance of imprudent stimulation or traction of the cranial nerve during surgery. From this series, linear contrast defects in the tumor indicate cranial nerve penetration, which is a useful finding for devising surgical strategies against posterior fossa meningiomas (Figure 1).

## 6. Conclusions and Future Directions

Various techniques for visualizing the vascular structure and cranial nerves related to skull base surgery in preoperative imaging are available, with each modality having advantages and disadvantages in terms of accuracy and complexity. It is necessary to evaluate each structure, tumor, vessel, and nerve using the appropriate modalities. Multimodal preoperative simulations that combine these modalities are also becoming possible [15,37]. Personalized 3D visualization technologies not only help understand the complicated anatomical skull base structures for each patient but also enable surgical simulations that are closer to real surgery using virtual reality, augmented reality, and three-dimensional printed models [83].

Artificial intelligence (AI)-based deep learning is likely to advance in the future for efficient surgical simulations. AI enables the accurate segmentation of surgical structures and optimizes preoperative planning [84]. Imaging analysis with AI aids in identifying critical structures from preoperative images, leading to more precise surgery. Moreover, AI may be able to consider things beyond the human experience by learning vast amounts of patient data. This could allow AI to perform objective assessments, potentially leading to accurate diagnoses of rare anatomical variations or detailed interpretations of intricate cerebrovascular flow patterns.

We expect to be able to identify all relevant structures from preoperative images, perform detailed surgical simulations, and develop comprehensive surgical strategies for skull base tumors. We believe that advances in medical imaging and AI technologies have the potential to significantly improve surgical planning and outcomes in complex skull base surgeries.

## Figures and Tables

**Table 1:** Previous reports of meningiomas and cranial nerve visualization.

Tumor Location	Authors	Year	Imaging Modality	Target Cranial Nerve	Identification Rate of Cranial Nerve in Preoperative Image, % (n)	Accuracy Rate Confirmed During the Surgery, % (n)
Parasellar	Sumida M	1998	SPGR	II	50.0% (7/14)	-
Parasellar	Saeki N	2002	Heavily T2	II	85.7% (6/7)	-
Parasellar	Ma J	2016	DTI	II	100% (3/3)	100% (2/2)
Parasellar	Zolal A	2017	DTI	II	50.0% (1/2)	100% (1/1)
Petroclival	Yang K	2017	FIESTA	VI	100% (1/1)	100% (1/1)
Cerebropontine angle	Hokamura M	2024	FACE	VII	50.0% (1/2)	100% (1/1)
Cerebropontine angle	Mikami T	2005	FIESTA + C	V, VI, VII, VIII, IX, X, XII	87.5% (7/8)	100% (7/7)
Petroclival	Ma J	2016	DTI	V, VI, VII, VIII	100% (3/3)	100% (3/3)
Petroclival	Yoshino M	2016	DTI	III, IV, V, VI	100% (1/1)	100% (1/1)
Posterior fossa	Behan B	2017	DTI	V, VII, VIII	100% (3/3)	-
Cerebropontine angle	Zolal A	2017	DTI	V, VII, VIII	100% (1/1)	100% (1/1)
Cerebropontine angle	Epprecht L	2019	DTI	VII, VIII	100% (1/1)	-
Cerebropontine angle	Churi ON	2019	DTI	V, VII, VIII	100% (2/2)	100% (2/2)
Posterior fossa	Szmuda T	2020	DTI	VII	100% (4/4)	100% (4/4)

C: contrast, DTI: diffusion tensor imaging, FACE: fast asymmetric spin echo, FIESTA: fast imaging employing steady-state acquisition, and SPGR: spoiled gradient recalled acquisition in steady state.

**Table 2:** Previous reports of schwannomas and cranial nerve visualization.

Tumor Origin	Authors	Year	Imaging Modality	Target Cranial Nerve	Identification Rate of Cranial Nerve in Preoperative Image, % (n)	Accuracy Rate Confirmed During the Surgery, % (n)
Vestibular nerve	Mikami T	2005	FIESTA + C	VII, VIII	22.2% (2/9)	-
Vestibular nerve	Sartoretti-Schefer S	2000	T2 FSE	VII	9.1% (2/22)	-
Vestibular nerve	Hokamura M	2024	FACE	VII	36.4% (4/11)	100% (4/4)
Vestibular nerve	Taoka T	2006	DTI	VII	87.5% (7/8)	83.3% (5/6)
Vestibular nerve	Gerganov VM	2011	DTI	VII	100% (22/22)	90.1% (20/22)
Vestibular nerve	Wei PH	2015	DTI	VII	100% (23/23)	95.5% (21/22)
Vestibular nerve	Ma J	2016	DTI	VII	88.9% (8/9)	100% (8/8)
Vestibular nerve	Song F	2016	DTI	VII	93.3% (14/15)	92.9% (13/14)
Vestibular nerve	Behan B	2017	DTI	V, VII, VIII	100% (6/6)	100% (6/6)
Vestibular nerve	Zolal A	2017	DTI	VII, VIII	100% (2/2)	100% (2/2)
Vestibular nerve	Zhang Y	2017	DTI	VII	100% (30/30)	100% (29/29)
Vestibular nerve	Li H	2017	DTI	VII	94.7% (18/19)	94.4% (17/18)
Vestibular nerve	Churi ON	2019	DTI	VII	100% (32/32)	100% (32/32)
Vestibular nerve	Szmuda T	2020	DTI	VII	90.6% (29/32)	89.7% (26/29)
Vestibular nerve	Zhang Y	2023	DTI	VII	90.0% (27/30)	92.6% (25/27)
Trigeminal nerve	Mikami T	2005	FIESTA + C	V	0.0% (0/2)	-
Trigeminal nerve	Behan B	2017	DTI	V	100% (1/1)	100% (1/1)
Trigeminal nerve	Churi ON	2019	DTI	V	100% (2/2)	50.0% (1/2)
Lower cranial nerve	Mikami T	2005	FIESTA + C	IX, X, XI	100% (1/1)	-

C: contrast, DTI: diffusion tensor imaging, FACE: fast asymmetric spin echo, FIESTA: fast imaging employing steady-state acquisition, and FSE: fast spin echo.

**Table 3:** Characteristics and findings of patients with posterior fossa meningioma with cranial nerve contrast defects.

Case	Age	Sex	Attachment	Contrast Defect	Intraoperative Findings of CNs	Pathology
1	47	F	Petrous	LCNs	Penetrated	Meningothelial
2	75	F	Foramen magnum	LCNs	Penetrated	Transitional
3	48	F	Petroclival	V	Penetrated	Meningothelial
				VI	Not observed	
				VII-VIII	Not observed	
4	78	M	Petroclival	V	Penetrated	Meningothelial
				VI	Penetrated	
5	70	M	Petroclival	V	Penetrated	Clear cell
6	48	F	Petrotentorial	V	Penetrated	Meningothelial
7	74	F	Petrotentorial	V	Penetrated	Transitional
				VI	Not observed	
8	45	F	Jugular tubercle	LCNs	Penetrated	Meningothelial
9	61	F	Petroclival	VI	Penetrated	Meningothelial
10	35	F	Petrous	LCN	Penetrated	Meningothelial

CN: cranial nerve, F: female, LCN: lower cranial nerve, M: male.

## Data Availability

The data presented in this study are available upon request from the corresponding author.

## References

[1] Anaizi A.N., Gantwerker E.A., Pensak M.L., Theodosopoulos P.V. (2014). Facial Nerve Preservation Surgery for Koos Grade 3 and 4 Vestibular Schwannomas. Neurosurgery.

[2] Ichinose T., Goto T., Ishibashi K., Takami T., Ohata K. (2010). The Role of Radical Microsurgical Resection in Multimodal Treatment for Skull Base Meningioma. J. Neurosurg..

[3] Scheer M., Simmermacher S., Prell J., Leisz S., Scheller C., Mawrin C., Strauss C., Rampp S. (2023). Recurrences and Progression Following Microsurgery of Vestibular Schwannoma. Front. Surg..

[4] Boari N., Gagliardi F., Cavalli A., Gemma M., Ferrari L., Riva P., Mortini P. (2016). Skull Base Chordomas: Clinical Outcome in a Consecutive Series of 45 Patients with Long-Term Follow-up and Evaluation of Clinical and Biological Prognostic Factors. J. Neurosurg..

[5] Jahangiri A., Chin A.T., Wagner J.R., Kunwar S., Ames C., Chou D., Barani I., Parsa A.T., McDermott M.W., Benet A. (2015). Factors Predicting Recurrence After Resection of Clival Chordoma Using Variable Surgical Approaches and Radiation Modalities. Neurosurgery.

[6] Borghei-Razavi H., Tomio R., Fereshtehnejad S.-M., Shibao S., Schick U., Toda M., Yoshida K., Kawase T. (2016). Pathological Location of Cranial Nerves in Petroclival Lesions: How to Avoid Their Injury During Anterior Petrosal Approach. J. Neurol. Surg. B Skull Base.

[7] Tomio R., Horiguchi T., Shibao S., Tamura R., Yoshida K., Kawase T. (2024). Anterior Transpetrosal Approach and the Tumor Removal Rate, Postoperative Neurological Changes, and Complications: Experience in 274 Cases over 33 Years. J. Neurosurg..

[8] Yang K., Ikawa F., Onishi S., Kolakshyapati M., Takeda M., Yamaguchi S., Ishifuro M., Akiyama Y., Morishige M., Kurisu K. (2017). Preoperative Simulation of the Running Course of the Abducens Nerve in a Large Petroclival Meningioma: A Case Report and Literature Review. Neurosurg. Rev..

[9] Lin J., Zhou Z., Guan J., Zhu Y., Liu Y., Yang Z., Lin B., Jiang Y., Quan X., Ke Y. (2018). Using Three-Dimensional Printing to Create Individualized Cranial Nerve Models for Skull Base Tumor Surgery. World Neurosurg..

[10] Sampath P., Rini D., Long D.M. (2000). Microanatomical Variations in the Cerebellopontine Angle Associated with Vestibular Schwannomas (Acoustic Neuromas): A Retrospective Study of 1006 Consecutive Cases. J. Neurosurg..

[11] Kurtsoy A., Menku A., Tucer B., Oktem I.S., Akdemir H. (2005). Neuronavigation in Skull Base Tumors. Minim. Invasive Neurosurg..

[12] Jödicke A., Ottenhausen M., Lenarz T. (2018). Clinical Use of Navigation in Lateral Skull Base Surgery: Results of a Multispecialty National Survey among Skull Base Surgeons in Germany. J. Neurol. Surg. B Skull Base.

[13] Matsushima K., Kohno M., Sakamoto H., Ichimasu N., Nakajima N. (2022). Intraoperative Continuous Neuromonitoring for Vestibular Schwannoma Surgery: Real-Time, Quantitative, and Functional Evaluation. World Neurosurg..

[14] Prado M.B., Kubota Y. (2024). Utility and Prognostic Value of Intraoperative Blink Reflex in Trigeminal or Facial Nerve Monitoring in Skull Base Surgeries: A Systematic Review. World Neurosurg..

[15] Jian Z.-H., Chen P., Li Y., Liao C.-C., Yi X.-F., Zhan R.-G., Chen G. (2024). Surgical Management of Complex Skull Base Tumor Using Preoperative Multimodal Image Fusion Technology. J. Craniofacial Surg..

[16] Gardner P.A., Tormenti M.J., Pant H., Fernandez-Miranda J.C., Snyderman C.H., Horowitz M.B. (2013). Carotid Artery Injury during Endoscopic Endonasal Skull Base Surgery: Incidence and Outcomes: Incidence and Outcomes. Neurosurgery.

[17] Van Der Veken J., Simons M., Mulcahy M.J., Wurster C., Harding M., Van Velthoven V. (2022). The Surgical Management of Intraoperative Intracranial Internal Carotid Artery Injury in Open Skull Base Surgery—A Systematic Review. Neurosurg. Rev..

[18] Origitano T.C., Al-Mefty O., Leonetti J.P., DeMonte F., Reichman O.H. (1994). Vascular Considerations and Complications in Cranial Base Surgery. Neurosurgery.

[19] Adachi K., Murayama K., Hayakawa M., Hasegawa M., Muto J., Nishiyama Y., Ohba S., Hirose Y. (2021). Objective and Quantitative Evaluation of Angiographic Vascularity in Meningioma: Parameters of Dynamic Susceptibility Contrast-Perfusion-Weighted Imaging as Clinical Indicators of Preoperative Embolization. Neurosurg. Rev..

[20] Yoshida K., Akiyama T., Takahashi S., Miwa T., Horiguchi T., Sasaki H., Toda M. (2021). Cone-Beam Computed Tomography Fusion Technique for Vascular Assessment of Skull Base Meningiomas. World Neurosurg..

[21] Shibao S., Toda M., Orii M., Fujiwara H., Yoshida K. (2016). Various Patterns of the Middle Cerebral Vein and Preservation of Venous Drainage During the Anterior Transpetrosal Approach. J. Neurosurg..

[22] Adachi K., Hasegawa M., Hirose Y. (2017). Evaluation of Venous Drainage Patterns for Skull Base Meningioma Surgery. Neurol. Med. Chir..

[23] Arima H., Watanabe Y., Tanoue Y., Morisako H., Kawakami T., Ichinose T., Goto T. (2023). Angiographic Evaluation of the Feeding Artery in Skull Base Meningioma. J. Clin. Med. Res..

[24] Tsuchiya K., Hachiya J., Mizutani Y., Yoshino A. (1996). Three-Dimensional Helical CT Angiography of Skull Base Meningiomas. Am. J. Neuroradiol..

[25] Bi W.L., Brown P.A., Abolfotoh M., Al-Mefty O., Mukundan S., Dunn I.F. (2015). Utility of Dynamic Computed Tomography Angiography in the Preoperative Evaluation of Skull Base Tumors. J. Neurosurg..

[26] Uetani H., Akter M., Hirai T., Shigematsu Y., Kitajima M., Kai Y., Yano S., Nakamura H., Makino K., Azuma M. (2013). Can 3T MR Angiography Replace DSA for the Identification of Arteries Feeding Intracranial Meningiomas?. Am. J. Neuroradiol..

[27] Hiramatsu M., Sugiu K., Hishikawa T., Haruma J., Takahashi Y., Murai S., Nishi K., Yamaoka Y., Shimazu Y., Fujii K. (2020). Detailed Arterial Anatomy and Its Anastomoses of the Sphenoid Ridge and Olfactory Groove Meningiomas with Special Reference to the Recurrent Branches from the Ophthalmic Artery. Am. J. Neuroradiol..

[28] Yoon N., Shah A., Couldwell W.T., Kalani M.Y.S., Park M.S. (2018). Preoperative Embolization of Skull Base Meningiomas: Current Indications, Techniques, and Pearls for Complication Avoidance. Neurosurg. Focus.

[29] Hirohata M., Abe T., Fujimura N., Takeuchi Y., Shigemori M. (2006). Preoperative Embolization of Brain Tumor with Pial Artery or Dural Branch of Internal Carotid Artery as Feeding Artery. Interv. Neuroradiol..

[30] Sumi K., Otani N., Mori F., Yamamuro S., Oshima H., Yoshino A. (2021). Venous Hypertension Caused by a Meningioma Involving the Sigmoid Sinus: Case Report. BMC Neurol..

[31] Watanabe T., Igarashi T., Fukushima T., Yoshino A., Katayama Y. (2013). Anatomical Variation of Superior Petrosal Vein and Its Management during Surgery for Cerebellopontine Angle Meningiomas. Acta Neurochir..

[32] Sindou M.P., Alvernia J.E. (2006). Results of Attempted Radical Tumor Removal and Venous Repair in 100 Consecutive Meningiomas Involving the Major Dural Sinuses. J. Neurosurg..

[33] Yamano A., Matsuda M., Kohzuki H., Ishikawa E. (2024). Impact of Superficial Middle Cerebral Vein Compression on Peritumoral Brain Edema of the Sphenoid Wing Meningioma. Clin. Neurol. Neurosurg..

[34] Teranishi Y., Kohno M., Sora S., Sato H. (2014). Determination of the Keyhole Position in a Lateral Suboccipital Retrosigmoid Approach. Neurol. Med. Chir..

[35] Jian Z.-H., Sheng M.-F., Li J.-Y., Li Y., Weng Z.-J., Chen G. (2022). Precise Localization in Craniotomy with a Retrosigmoid Keyhole Approach: Microsurgical Anatomy and Clinical Study. Front. Surg..

[36] Feigl G.C., Krischek B., Ritz R., Thaher F., Marquardt J.S., Hirt B., Korn A., Schumann M., Tatagiba M., Ebner F.H. (2014). Evaluation of a 3-Dimensional Voxel-Based Neuronavigation System with Perspective Image Rendering for Keyhole Approaches to the Skull Base: An Anatomical Study. World Neurosurg..

[37] Oishi M., Fukuda M., Ishida G., Saito A., Hiraishi T., Fujii Y. (2011). Presurgical Simulation with Advanced 3-Dimensional Multifusion Volumetric Imaging in Patients with Skull Base Tumors. Neurosurgery.

[38] Sato M., Tateishi K., Murata H., Kin T., Suenaga J., Takase H., Yoneyama T., Nishii T., Tateishi U., Yamamoto T. (2018). Three-Dimensional Multimodality Fusion Imaging as an Educational and Planning Tool for Deep-Seated Meningiomas. Br. J. Neurosurg..

[39] Chakeres D.W., Kapila A. (1983). Brainstem and Related Structures: Normal CT Anatomy Using Direct Longitudinal Scanning with Metrizamide Cisternography. Radiology.

[40] Mamata Y., Muro I., Matsumae M., Komiya T., Toyama H., Tsugane R., Sato O. (1998). Magnetic Resonance Cisternography for Visualization of Intracisternal Fine Structures. J. Neurosurg..

[41] Seitz J., Held P., Strotzer M., Völk M., Nitz W.R., Dorenbeck U., Stamato S., Feuerbach S. (2002). MR Imaging of Cranial Nerve Lesions Using Six Different High-Resolution T1- and T2(*)-Weighted 3D and 2D Sequences. Acta Radiol..

[42] Ciftci E., Anik Y., Arslan A., Akansel G., Sarisoy T., Demirci A. (2004). Driven Equilibrium (Drive) MR Imaging of the Cranial Nerves V-VIII: Comparison with the T2-Weighted 3D TSE Sequence. Eur. J. Radiol..

[43] Yousry I., Camelio S., Schmid U.D., Horsfield M., Wiesmann M., Brückmann H., Yousry T. (2000). Visualization of Cranial Nerves I–XII: Value of 3D CISS and T2-Weighted FSE Sequences. Eur. Radiol..

[44] Aydın H., Altın E., Dilli A., Sipahioğlu S., Hekimoğlu B. (2011). Evaluation of Jugular Foramen Nerves by Using B-FFE, T2-Weighted DRIVE, T2-Weighted FSE and Post-Contrast T1-Weighted MRI Sequences. Diagn. Interv. Radiol..

[45] Moon W.-J., Roh H.G., Chung E.C. (2009). Detailed MR Imaging Anatomy of the Cisternal Segments of the Glossopharyngeal, Vagus, and Spinal Accessory Nerves in the Posterior Fossa: The Use of 3D Balanced Fast-Field Echo MR Imaging. Am. J. Neuroradiol..

[46] Yagi A., Sato N., Taketomi A., Nakajima T., Morita H., Koyama Y., Aoki J., Endo K. (2005). Normal Cranial Nerves in the Cavernous Sinuses: Contrast-Enhanced Three-Dimensional Constructive Interference in the Steady State MR Imaging. Am. J. Neuroradiol..

[47] Özgür A., Esen K., Kara E., Temel G.O. (2017). Visualization of the Abducens Nerve in Its Petroclival Segment Using Contrast-Enhanced FIESTA MRI: The Size of the Petroclival Venous Confluence Affects Detectability. Clin. Neuroradiol..

[48] Blitz A.M., Macedo L.L., Chonka Z.D., Ilica A.T., Choudhri A.F., Gallia G.L., Aygun N. (2014). High-Resolution CISS MR Imaging with and Without Contrast for Evaluation of the Upper Cranial Nerves: Segmental Anatomy and Selected Pathologic Conditions of the Cisternal Through Extraforaminal Segments. Neuroimaging Clin..

[49] Mori S., van Zijl P.C.M. (2002). Fiber Tracking: Principles and Strategies—A Technical Review. NMR Biomed..

[50] Joo W., Rhoton A.L. (2015). Microsurgical Anatomy of the Trochlear Nerve. Clin. Anat..

[51] Hodaie M., Quan J., Chen D.Q. (2010). In Vivo Visualization of Cranial Nerve Pathways in Humans Using Diffusion-Based Tractography. Neurosurgery.

[52] Yoshino M., Abhinav K., Yeh F.-C., Panesar S., Fernandes D., Pathak S., Gardner P.A., Fernandez-Miranda J.C. (2016). Visualization of Cranial Nerves Using High-Definition Fiber Tractography. Neurosurgery.

[53] Ostrom Q.T., Gittleman H., Truitt G., Boscia A., Kruchko C., Barnholtz-Sloan J.S. (2018). CBTRUS Statistical Report: Primary Brain and Other Central Nervous System Tumors Diagnosed in the United States in 2011–2015. Neuro-Oncology.

[54] Paldor I., Awad M., Sufaro Y.Z., Kaye A.H., Shoshan Y. (2016). Review of Controversies in Management of Non-Benign Meningioma. J. Clin. Neurosci..

[55] Sumida M., Arita K., Migita K., Iida K., Kurisu K., Uozumi T. (1998). Demonstration of the Optic Pathway in Sellar/Juxtasellar Tumours with Visual Disturbance on MR Imaging. Acta Neurochir..

[56] Saeki N., Murai H., Kubota M., Fujimoto N., Iuchi T., Yamaura A., Sunami K. (2002). Heavily T2 Weighted MR Images of Anterior Optic Pathways in Patients with Sellar and Parasellar Tumours—Prediction of Surgical Anatomy. Acta Neurochir..

[57] Ma J., Su S., Yue S., Zhao Y., Li Y., Chen X., Ma H. (2016). Preoperative Visualization of Cranial Nerves in Skull Base Tumor Surgery Using Diffusion Tensor Imaging Technology. Turk. Neurosurg..

[58] Zolal A., Sobottka S.B., Podlesek D., Linn J., Rieger B., Juratli T.A., Schackert G., Kitzler H.H. (2017). Comparison of Probabilistic and Deterministic Fiber Tracking of Cranial Nerves. J. Neurosurg..

[59] Hokamura M., Uetani H., Hamasaki T., Nakaura T., Morita K., Yamashita Y., Kitajima M., Sugitani A., Mukasa A., Hirai T. (2024). Effect of Deep Learning-Based Reconstruction on High-Resolution Three-Dimensional T2-Weighted Fast Asymmetric Spin-Echo Imaging in the Preoperative Evaluation of Cerebellopontine Angle Tumors. Neuroradiology.

[60] Mikami T., Minamida Y., Yamaki T., Koyanagi I., Nonaka T., Houkin K. (2005). Cranial Nerve Assessment in Posterior Fossa Tumors with Fast Imaging Employing Steady-State Acquisition (FIESTA). Neurosurg. Rev..

[61] Epprecht L., Kozin E.D., Piccirelli M., Kanumuri V.V., Tarabichi O., Remenschneider A., Barker F.G., McKenna M.J., Huber A.M., Cunnane M.E. (2019). Super-Resolution Diffusion Tensor Imaging for Delineating the Facial Nerve in Patients with Vestibular Schwannoma. J. Neurol. Surg. B Skull Base.

[62] Churi O.N., Gupta S., Misra B.K. (2019). Correlation of Preoperative Cranial Nerve Diffusion Tensor Tractography with Intraoperative Findings in Surgery of Cerebellopontine Angle Tumors. World Neurosurg..

[63] Szmuda T., Słoniewski P., Ali S., Pereira P.M.G., Pacholski M., Timemy F., Sabisz A., Szurowska E., Kierońska S. (2020). Reliability of Diffusion Tensor Tractography of Facial Nerve in Cerebello-Pontine Angle Tumours. Neurol. Neurochir. Pol..

[64] Behan B., Chen D.Q., Sammartino F., DeSouza D.D., Wharton-Shukster E., Hodaie M. (2017). Comparison of Diffusion-Weighted MRI Reconstruction Methods for Visualization of Cranial Nerves in Posterior Fossa Surgery. Front. Neurosci..

[65] Agarwal A. (2015). Intracranial Trigeminal Schwannoma. Neuroradiol. J..

[66] Aftahy A.K., Groll M., Barz M., Bernhardt D., Combs S.E., Meyer B., Negwer C., Gempt J. (2021). Surgical Management of Jugular Foramen Schwannomas. Cancers.

[67] Myrseth E., Møller P., Pedersen P.-H., Lund-Johansen M. (2009). Vestibular Schwannoma: Surgery or Gamma Knife Radiosurgery? A Prospective, Nonrandomized Study. Neurosurgery.

[68] Hasegawa T., Kida Y., Kobayashi T., Yoshimoto M., Mori Y., Yoshida J. (2005). Long-Term Outcomes in Patients with Vestibular Schwannomas Treated Using Gamma Knife Surgery: 10-Year Follow Up. J. Neurosurg..

[69] Seol H.J., Kim C.-H., Park C.-K., Kim C.H., Kim D.G., Chung Y.-S., Jung H.-W. (2006). Optimal Extent of Resection in Vestibular Schwannoma Surgery: Relationship to Recurrence and Facial Nerve Preservation. Neurol. Med. Chir..

[70] Iwai Y., Ishibashi K., Watanabe Y., Uemura G., Yamanaka K. (2015). Functional Preservation after Planned Partial Resection Followed by Gamma Knife Radiosurgery for Large Vestibular Schwannomas. World Neurosurg..

[71] Dong C.C.J., Macdonald D.B., Akagami R., Westerberg B., Alkhani A., Kanaan I., Hassounah M. (2005). Intraoperative Facial Motor Evoked Potential Monitoring with Transcranial Electrical Stimulation during Skull Base Surgery. Clin. Neurophysiol..

[72] Amano M., Kohno M., Nagata O., Taniguchi M., Sora S., Sato H. (2011). Intraoperative Continuous Monitoring of Evoked Facial Nerve Electromyograms in Acoustic Neuroma Surgery. Acta Neurochir..

[73] Sartoretti-Schefer S., Kollias S., Valavanis A. (2000). Spatial Relationship between Vestibular Schwannoma and Facial Nerve on Three-Dimensional T2-Weighted Fast Spin-Echo MR Images. Am. J. Neuroradiol..

[74] Taoka T., Hirabayashi H., Nakagawa H., Sakamoto M., Myochin K., Hirohashi S., Iwasaki S., Sakaki T., Kichikawa K. (2006). Displacement of the Facial Nerve Course by Vestibular Schwannoma: Preoperative Visualization Using Diffusion Tensor Tractography. J. Magn. Reson. Imaging Off. J. Int. Soc. Magn. Reson. Med..

[75] GerGanoV V.M., GiorDano M., SaMii M., Samii A. (2011). Diffusion Tensor Imaging-Based Fiber Tracking for Prediction of the Position of the Facial Nerve in Relation to Large Vestibular Schwannomas. J. Neurosurg..

[76] Wei P.-H., Qi Z.-G., Chen G., Hu P., Li M.-C., Liang J.-T., Guo H.-C., Ling F., Bao Y.-H. (2015). Identification of Cranial Nerves near Large Vestibular Schwannomas Using Superselective Diffusion Tensor Tractography: Experience with 23 Cases. Acta Neurochir..

[77] Song F., Hou Y., Sun G., Chen X., Xu B., Huang J.H., Zhang J. (2016). In Vivo Visualization of the Facial Nerve in Patients with Acoustic Neuroma Using Diffusion Tensor Imaging-Based Fiber Tracking. J. Neurosurg..

[78] Zhang Y., Mao Z., Wei P., Jin Y., Ma L., Zhang J., Yu X. (2017). Preoperative Prediction of Location and Shape of Facial Nerve in Patients with Large Vestibular Schwannomas Using Diffusion Tensor Imaging-Based Fiber Tracking. World Neurosurg..

[79] Li H., Wang L., Hao S., Li D., Wu Z., Zhang L., Zhang J. (2017). Identification of the Facial Nerve in Relation to Vestibular Schwannoma Using Preoperative Diffusion Tensor Tractography and Intraoperative Tractography-Integrated Neuronavigation System. World Neurosurg..

[80] Zhang Y., Ge H., Xu M., Mei W. (2023). Significance of Preoperative Nerve Reconstruction Using Diffusion Tensor Imaging Tractography for Facial Nerve Protection in Vestibular Schwannoma. J. Korean Neurosurg. Soc..

[81] Chen D.Q., Quan J., Guha A., Tymianski M., Mikulis D., Hodaie M. (2011). Three-Dimensional in Vivo Modeling of Vestibular Schwannomas and Surrounding Cranial Nerves with Diffusion Imaging Tractography. Neurosurgery.

[82] Amemiya S., Aoki S., Ohtomo K. (2009). Cranial Nerve Assessment in Cavernous Sinus Tumors with Contrast-Enhanced 3D Fast-Imaging Employing Steady-State Acquisition MR Imaging. Neuroradiology.

[83] Isikay I., Cekic E., Baylarov B., Tunc O., Hanalioglu S. (2024). Narrative Review of Patient-Specific 3D Visualization and Reality Technologies in Skull Base Neurosurgery: Enhancements in Surgical Training, Planning, and Navigation. Front. Surg..

[84] Neves C.A., Tran E.D., Blevins N.H., Hwang P.H. (2021). Deep Learning Automated Segmentation of Middle Skull-Base Structures for Enhanced Navigation. Int. Forum Allergy Rhinol..

